# Brain Regulates Neuronal Activity Directly through the Heartbeat: A New Pathway of Heart-Brain Interaction

**DOI:** 10.14336/AD.2024.1083

**Published:** 2025-11-12

**Authors:** Zhitian Wang, Junjian Zhang, Qing-Guo Ren

**Affiliations:** ^1^Department of Neurology, ZhongDa Hospital Affiliated to Southeast University, Nanjing, Jiangsu, China.; ^2^Zhongnan Hospital of Wuhan University, Wuhan, Hubei, China.; ^3^School of Medicine, Southeast University, Nanjing, Jiangsu, China.

**Dear Editor,**

The senses of vision, smell, hearing, and others continuously send external information to the brain, enabling us to perceive changes in our environment [1]. In addition to the exteroception, interoception is sometimes overlooked as an additional sense. We also have a variety of information within the body, that needs to be submitted to the brain in time for processing, such as heartbeat, respiration, and blood pressure changes [2]. Nervous electrical oscillations form the basis of the brain's information processing, respiration, heartbeat, and other internal sensory activities, which can, in turn, regulate these neural oscillations [3]. The mechanism by which interoception senses internal body signals and transmits the body's state to the brain, is one of the key scientific questions in neuroscience that needs to be addressed today.

**Figure 1.**
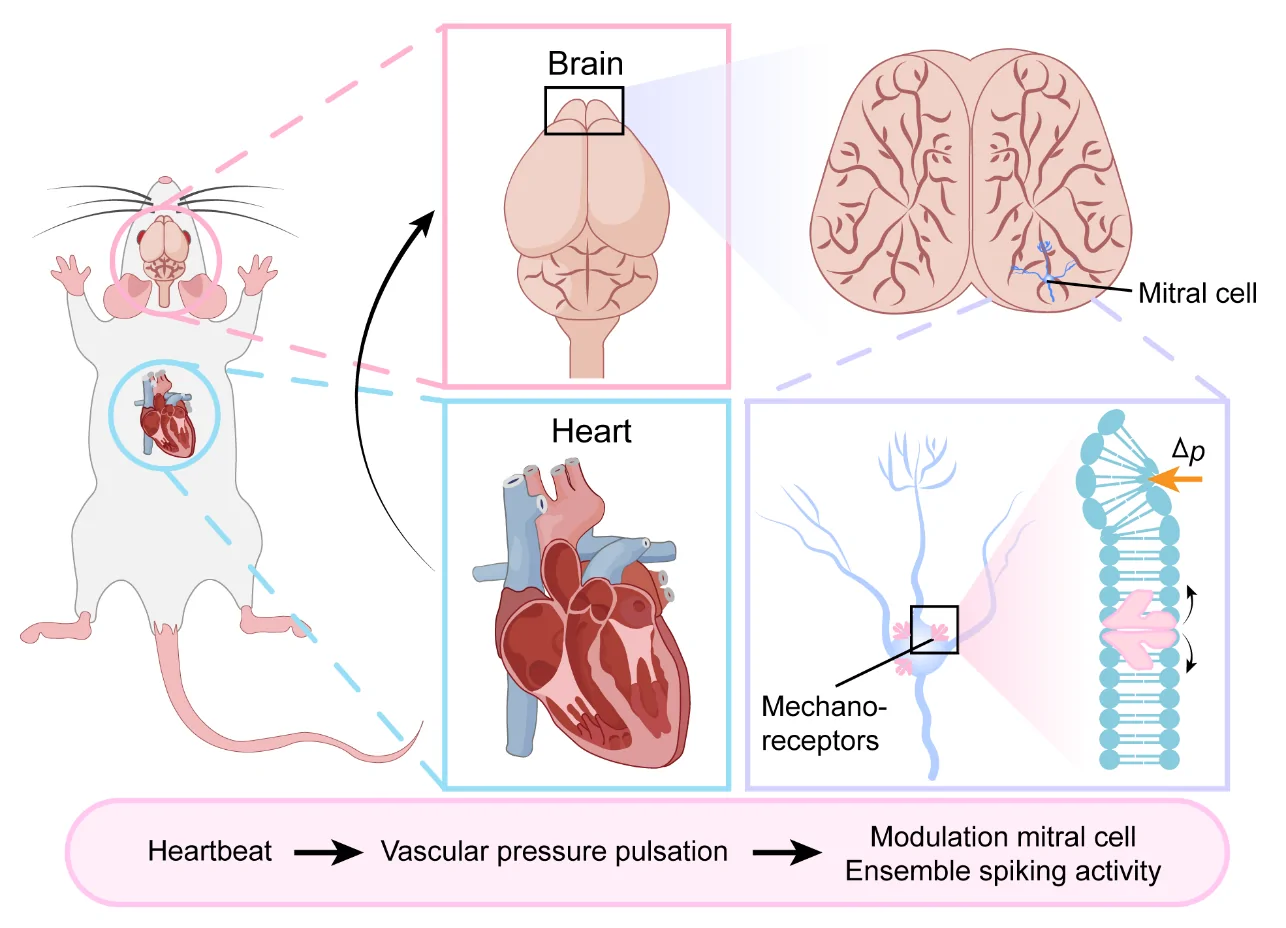
**Diagram of the brain regulating neuronal activity directly through the heartbeat**. Jammal Salameh *et al*. developed a semi-complete rat olfactory bulb-brain perfusion system to reduce olfactory stimulation by breathing. Heartbeat-associated vascular blood pressure pulsations (Δ*p*) caused the opening of mechanosensitive ion channels on mitral cells in the brain’s olfactory bulb, leading to spontaneous neuronal spiking activity.

Fortunately, a recent study published in *Science* found that spontaneous slow oscillations have occurred in the rat olfactory bulb local field potential, even in the absence of respiration [4]. This phenomenon attracted the keen interest of Jammal Salameh *et al*. They further identified a subpopulation of neurons within the olfactory bulb that could directly sense the vascular blood pressure pulsations associated with the heartbeat. By constructing a semi-intact preparation of the rat olfactory bulb, they found for the first time that mitral cells in the olfactory bulb could directly sense pressure fluctuations and change their neuroexcitatory rhythms through mechanosensitive ion channels, and their response rate (~20 ms) was much faster than the classical way of sensing blood pressure pulsation changes in the aortic sinus and carotid sinus (Fig. 1). Jammal Salameh *et al*. found mechanosensation was to be another interesting communication pathway, in addition to the pathways already described (neural, hormonal, etc.). Mechanosensation has been previously described as a mechanism related to touch, pain, and intestinal motility, and it has obvious connections to interoception. Thus, a more rapid interoception mechanism may exist to directly modulate central neuronal activity.

Jammal Salameh *et al*. developed a semi-intact rat nose-brain preparation (NBP) without respiratory stimulation of olfaction in a pre-existing effort to study the mechanisms of local neural oscillations in restricted conditions. The NBP was based on the perfusion of artificial cerebrospinal fluid via a peristaltic pump to generate pulsatile pressure within the cerebrovascular system, and adjusting the perfusion pressure by increasing the frequency of the peristaltic pump to conform to the heartbeat-induced physiological range of intracranial and cerebrovascular pressures [5]. In the NBP system, spontaneous slow oscillations were detectable in the olfactory bulb local field potential (LFP). To investigate whether neural electrical oscillations in the olfactory bulb were related to the blood pressure pulsations, Jammal Salameh *et al*. used NBP to monitor the electrical activity of cells in all layers of the olfactory bulb. The results showed that LFP oscillations with frequencies ranging from 1.5 to 4 Hz were recorded only in the mitral cells of the olfactory bulb and found that the blood pressure pulsations generated by the peristaltic pump corresponded to the fundamental frequency of the LFP oscillations, which essentially ceased in the mitral cells after the addition of an extra device in the perfusion circuit to mimic the elastic properties of the vascular system to reduce the blood pressure pulsations. The above results suggested that blood pressure pulsations were responsible for the generation of LFP oscillations in mitral cells.

To rule out signal artifacts caused by small arterial pulsations close to the recording electrodes, Jammal Salameh *et al*. tested the neurogenic hypothesis of LFP oscillations by interfering with tissue viability in two ways. One was to reduce the peristaltic pump frequency from 15-30 rpm to 3 rpm, and the other was to change the perfused aerobic artificial cerebrospinal fluid to anaerobic artificial cerebrospinal fluid, by which neuronal activity was inhibited. The results showed that the LFP oscillations recorded in the olfactory bulb almost completely disappeared after inhibition of neuronal activity, which demonstrated that the LFP oscillations were not signaling artifacts caused by small arterial pulsations, but were of neural origin. So how do mitral cells sense this blood pressure pulsation? The first thing that came to mind was the mechanosensitive ion channels, the Piezo channel family. It is a mechanically gated cation channel class that senses touch, pain, visceral sensation, and cardiovascular activity in milliseconds [6]. Previous studies have also reported Piezo2 expression in mouse mitral valve cells [7]. Since there was a lack of selective antagonists for Piezo2, Jammal Salameh *et al*. chose to locally inject the cationic mechanosensitive ion channel antagonist GsMTx4 to interfere with a variety of mechanosensitive ion channels [8], including Piezo1/2 and transient receptor potential subfamily C (TRPC) channels, and the results showed that LFP oscillations were completely inhibited by capitalism, whereas the TRPC channel selective antagonist SKF 96365 had no significant effect on LFP oscillations. These results suggested that the perception of blood pressure pulsations by mitral cells was likely to be mediated by the Piezo family.

Due to differences in the gating properties of Piezo1 and Piezo2, Piezo2 is more sensitive to increases in pressure than decreases in pressure, whereas Piezo1 is sensitive to both increases and decreases in pressure, and there will be differences in the LFP waveforms of the two [9]. After a detailed analysis of the LFP waveforms, Jammal Salameh *et al*. concluded that Piezo2 was theoretically more consistent with the LFP waveforms produced by mitral cells sensing blood pressure pulsations. Finally, they further tested in vivo whether cerebral blood pressure pulsations modulate spontaneous neuronal activity in the olfactory bulb by recording heartbeat, respiration, and olfactory bulb electrical activity in awake mice, and found that both respiratory activity and heartbeat modulate olfactory bulb neuronal activity. Approximately 15% of olfactory bulb neurons showed a significant increase in firing activity within 20 ms after the heartbeat, an effect that was much weaker than the known coupling of neuronal activity to respiratory rhythms, and heartbeat-modulated neuronal activity was similarly observed in the hippocampus and cortex.

The above study prompts further reflection. Previous studies indicate internal physiological signals, such as blood pressure fluctuations and temperature changes, can significantly influence the excitability and function of neurons by affecting the activity of mechanosensitive ion channels [10]. This means that neurons respond to changes in the external environment and perceive and adjust their physiological state to adapt to internal and external stimuli. The interaction between internal signals and external stimuli may lead to the plasticity of neural networks, which in turn affects learning, memory, and behavior [11]. The brain achieves more efficient signal processing and information integration in this manner. Understanding how internal physiological signals impact neuronal activity can help us better comprehend how the brain integrates internal and external information. This integration capability is crucial for maintaining physiological homeostasis, responding to environmental changes, and making adaptive responses in complex situations.

Cardio-cerebral interaction is a complex, multi-layered process involving various mechanisms such as hemodynamics, autonomic nervous regulation, endocrine signaling, and neural signal synchronization [12, 13]. In addition, previous studies have reported different mechanisms of neurovascular coupling, such as neurons releasing signals through synapses to activate surrounding vascular smooth muscle and endothelial cells, thereby regulating vasodilation [14]. Based on these mechanisms, the interaction between the heart and brain helps maintain physiological balance and plays a crucial role in responding to environmental changes, regulating emotions, and managing stress. Compared to the known mechanisms of cardio-cerebral interaction, the novelty of this study lies in the discovery that mechanosensation is another interesting communication pathway.

Mechanosensation has previously been described as a mechanism related to touch, pain, and gut motility, and is closely linked to interoception. Furthermore, the response time of this mechanosensation is much faster than the classical way of baroreceptors in the aortic arch and carotid sinus sensing blood pressure pulsation. Therefore, there may exist a more rapid interoceptive mechanism that directly regulates central neuronal activity.

Under normal physiological conditions, the natural fluctuations in blood pressure may subtly regulate neuronal activity through mechanosensitive ion channels [15]. This mechanism could play a crucial role in maintaining the stability of blood pressure and cardiovascular function. In conditions such as hypertension, hypotension, or other cardiovascular diseases, abnormal blood pressure fluctuations may lead to aberrant activation or inhibition of mechanosensitive ion channels [16]. This alteration could further affect neuronal excitability and, consequently, the functionality of neural networks in the brain. This mechanism might be closely associated with the pathological development of these diseases. Studies have found that patients with heart failure often exhibit significant fluctuations in blood pressure. Mechanosensitive ion channels may play an important role in sensing and responding to these fluctuations, thereby affecting the function of cardiomyocytes and endothelial cells [6, 17]. In the early development of atherosclerosis, hemodynamic changes may regulate endothelial cell function and inflammatory responses through mechanosensitive ion channels [18]. Exploring the potential impact of blood pressure dynamics on cardiovascular diseases not only aids in understanding the mechanisms of these diseases but may also drive the development of new diagnostic and therapeutic methods.

In the future, this new interoceptive mechanism may provide novel biomarkers for the early diagnosis of cardio-cerebral-related diseases such as hypertension, heart disease, and stroke. Due to the fast response of mechanosensation, this rapid feedback could offer more sensitive and timely detection compared to existing methods, helping doctors identify potential risks associated with cardio-cerebral interaction at an earlier stage. Moreover, by exploring ways to regulate this interoceptive mechanism, new therapeutic strategies may be developed to adjust the interaction between the heart and brain, thereby improving the prognosis of cardio-cerebral diseases.

In addition to the impact on cardiovascular and cerebrovascular diseases, internal physiological signals, such as fluctuations in blood pressure and temperature, may directly affect cognitive functions like attention, learning, and memory by modulating the excitability and plasticity of neurons. Research indicates that blood pressure fluctuations can impact the brain's efficiency in processing information, thereby influencing learning abilities and the formation of memories [19]. The connection between physiological signals and emotions is increasingly being recognized. Changes in internal signals can alter emotional states by affecting neurotransmitter release and neuronal activity. For example, elevated blood pressure may be associated with increased levels of anxiety or stress, while low blood pressure may be linked to depressive moods [20, 21]. The brain’s ability to integrate internal physiological signals and external stimuli is also crucial for adaptive behaviors. In the face of threats, physiological signals may trigger a rapid “fight or flight” response, while in a calm environment, the body’s physiological state may lead to more deliberate decision-making processes [22, 23].

There are several issues about the current work that need investigation further.

First, the use of a semi-intact NBP in this study facilitated the discovery of heartbeat-mediated pressure sensory modulation of neuronal activity in mitral cells. The absence of ascending inputs from cardiorespiratory control centers, neuromodulatory inputs, and the lack of respiratory-related physical sensory feedback all contributed to the demonstration of the observed pressure-pulse-induced modulation of neuronal activity in the mitral cells. However, the effective pressure pulse amplitude Δ*p* in the NBP in the vicinity of the mitral cells is unknown and there is no good way to measure it. In vivo, the extent to which heartbeat-induced coupling may be modulated via vascular tone modulators or possibly via astrocytes needs to be further explored in the future. The researchers constructed the NBP system to explore the specific mechanism of heartbeat regulation of neuronal activity. However, due to the lack of physiological environment and system interactions in in vitro models, these models may not fully reflect the complex physiological conditions in vivo. Furthermore, the researchers validated their findings through animal experiments, discovering that blood pressure pulsation also regulates spontaneous neuronal activity in the olfactory bulb of living mice. However, considering the significant differences between animals and humans, macaques and organoid models may provide a better explanation of this mechanism. This study offers a new perspective on the interaction of the heart-brain axis. Nonetheless, given the limitations of experimental methods and sample selection, future research should integrate multiple models to enhance the reliability of the study and its clinical translational potential.

Second, in the present study, blood pressure pulsation was found to modulate neuronal activity through mechanical ion channels in the olfactory bulb region, and heartbeat modulation of neuronal activity was similarly observed in the hippocampus and cortex. Hippocampal and cortical sites are highly associated with memory and cognition [24], and it is not clear whether heartbeat modulation of neuronal activity is also important in the hippocampus and cortex and what the specific mechanisms are. This issue seems equally interesting and deserves further exploration.

Finally, according to the somatic marker hypothesis, which suggests that emotional processing includes autonomic components that may be involved in respiration, gut motility, and the cardiovascular system, emotional states and conscious awareness are derived from internal sensations [25]. Previous studies have shown that increased optogenetic heart rate in mice forms anxious brain circuits [26]. There may be a central network of “heartbeat sentinel neurons” that play a crucial role in the internal sensory regulation of cognition, emotion, and autonomic states during arousal. This network may have important implications for brain function, prompting us to delve deeper into the links between heart and brain function, which is a direction for further research.

In the future, we can conduct in-depth basic research to explore how internal physiological signals specifically affect neuronal activity. Clinical studies can be conducted in different populations (such as patients with hypertension or mood disorders) to examine the impact of internal physiological signals on cognition and emotions. Additionally, longitudinal studies can be carried out to assess cognitive and emotional changes in individuals in response to fluctuations in physiological signals. Furthermore, we can investigate the possibility of improving cognitive and emotional states by modulating internal physiological signals through interventions such as medication or behavioral therapies.

Overall, the present study has deepened our understanding of interoception sense mechanisms within the brain by identifying a new mechanism by which blood pressure modulates neuronal excitability in the brain, namely, that mitral cells in the olfactory bulb can sense changes in blood pressure through mechanosensitive ion channels. This study demonstrates a vascular-neuronal interaction that is different from previous studies, providing new insights into heart-brain interactions and broadening our understanding of the connection between heart and brain activity.
